# The Association of Perceived Discrimination to Functional Limitation Among Immigrant Women Living in the U.S.

**DOI:** 10.1093/geroni/igaf122.2496

**Published:** 2025-12-31

**Authors:** Heather Hunter, Corey Nagel

**Affiliations:** University of Arkansas for Medical Sciences, Little Rock, Arkansas, United States; University of Arkansas for Medical Sciences, Little Rock, Arkansas, United States

## Abstract

The purpose of this study was to examine if perceived discrimination of middle aged and older (50+) immigrant women is associated with a decline in functional status. Immigrants moving to the United States face unique challenges in meeting their healthcare needs. They have increased risk for experiencing discrimination, which impacts health negatively. One area, discrimination, may effect functional ability, which is important to maintaining independence and involvement in community. Thus, we aimed to understand if discrimination has a relationship with functional status among a sample of middle aged and older (50+) immigrant women who participated in the Health and Retirement Study (HRS) during the period 2008-2018 (n = 2,773). We used Poisson regression with robust standard errors to examine the association between perceived discrimination (using responses from the Perceived Everyday Discrimination Scale) and functional status (counts of assistance required for ADLs and IADLs). In our sample, 36% reported discrimination and 17% reported frequent discrimination. Our models indicated that immigrant women who report experiencing any discrimination have 1.25 times greater number of limitations than women who are not experiencing discrimination (RR = 1.25; 95% CI = 1.04, 1.51; p = 0.020), and those immigrant women experiencing frequent discrimination have 1.39 times greater number of limitations than women who are not experiencing frequent discrimination (RR = 1.39; 95% CI = 1.12, 1.73; p; = 0.003). Understanding the relationship between discrimination and functional health is important for healthcare professionals to anticipate the needs of immigrant women and respond with appropriate interventions aimed at improving and sustaining functional health.

